# Differences in setting time of calcium silicate-based sealers under different test conditions

**DOI:** 10.1016/j.jds.2022.11.029

**Published:** 2022-12-10

**Authors:** Jina Koo, Sang Won Kwak, Hyeon-Cheol Kim

**Affiliations:** Department of Conservative Dentistry, Pusan National University School of Dentistry, Dental and Life Science Institute, Dental Research Institute, Yangsan, South Korea

**Keywords:** calcium silicate, Epoxy resin, Gypsum mold, Root canal sealer, Setting time, Standardization

## Abstract

**Background/purpose:**

Recently introduced calcium silicate-based bioceramic (CSBC) sealers require moisture for setting, thus this study aimed to compare the setting times of epoxy resin-and CSBC sealers under different test conditions.

**Materials and methods:**

Four CSBC sealers (CeraSeal, EndoSeal TCS, One-Fil, and Well-Root ST) were compared to an epoxy resin-based sealer (AH Plus). Each sealer was placed in a stainless-steel and gypsum molds on a glass slide. Sealer samples (n = 10 per group) were stored in an incubator at 95% humidity and 37 °C. A Gilmore needle with a total weight of 100 g and a 2.0-mm diameter were carefully placed vertically against the sealer, and the setting time was recorded when the needle no longer formed an indentation on the sealer surface. Statistical analysis was performed using two-way analysis of variance and Tukey parametric tests. The level of significance was set at 95%.

**Results:**

The setting time of all sealers in gypsum molds was significantly shorter than that in stainless-steel molds (*P* < 0.05). While AH Plus showed the longest setting time, EndoSeal TCS, One-Fil, and CeraSeal showed the shortest setting times when using gypsum molds among the five sealer types (*P* < 0.05).

**Conclusion:**

The results of this study indicate that CSBC sealers require moisture for setting; a lack of moisture results in a significant delay in setting time. Because the root canals contain moisture, it is necessary to experiment with the setting time of all types of sealers using gypsum molds to determine the biological condition of root canals.

## Introduction

Root canal treatment procedures include access opening, canal shaping and cleaning, canal filling, and building of core materials. Canal filling reflects the canal shaping and cleaning, and the quality of filling is an important determinant of the long-term success of root canal treatment.^1^ Canal obturation comprises a set of operative procedures performed in a specific sequence to achieve three-dimensional sealing.^2^ Obturation of the radicular space prevents infection (reinfection) of the root canal via leakage of microorganisms and their byproducts from the oral environment.^3^ Obturation reduces coronal leakage and bacterial contamination, seals the apex from periapical tissue fluids, and entombs the remaining irritants in the root canal.^4^ Most obturation techniques employ a core material, commonly a gutta-percha (GP) cone, and a sealer. The sealer can fill canal irregularities and dentinal tubules that cannot be filled with GP.^5^ This indicates that the sealer should act as a binding agent between the core material and root canal dentin.^6^ Regardless of the core material, a sealer is essential for every canal filling technique, and helps achieve a fluid-tight seal.^4^

Many types of sealers are generally used in dental clinics. The most well-known sealers are zinc-oxide-eugenol, Ca(OH)_2_, glass ionomer, resin-based epoxy resin or methacrylate resin, as well as the recently introduced calcium silicate-based bioceramic (CSBC) sealers.^4^ The well-documented biocompatibility and bioactivity of di- and tricalcium silicate cements are also properties attributed to modern CSBC sealers.^7^ CSBC sealers gained notoriety as an outgrowth of the popularity of mineral trioxide aggregate (MTA) materials, a water-setting hydraulic powder used for various surgical and vital pulp therapy treatments.^8^, ^9^, ^10^ This type of root canal sealer is attractive because of the high bioactivity of MTA-type materials, and because of their hydrophilicity.^11^, ^12^, ^13^ Recently, paste-based techniques using GP master cones and CSBC sealers have gained increasing interest.

In previous studies, the physical properties of sealers were evaluated for flow, setting time, radiopacity, dimensional stability, PH change, and solubility.^2^^,^^13^, ^14^, ^15^ Among them, setting time has an impact on the working time and clinical performance of endodontic sealers, while moisture content may affect the setting time values and adversely affect some properties, such as microhardness.^16^ In addition, since setting time provides sufficient consistency to completely fill the root canal system, the setting time of the sealer is important for apical sealing. A long setting time should be avoided because of the possible cytotoxic effect of the unset sealer materials contacting the periapical tissues.^17^ However, several studies have reported that CSBC sealers have a very long setting time and do not set when measuring the setting time using stainless-steel (SS) mold.^15^^,^^16^ As such, it is necessary to investigate whether measuring the setting time of sealers using SS molds can result in errors.

According to the recent ISO 6876/2012 standards, an SS mold with an internal diameter of 10 mm and a height of 2 mm should be used for sealers that do not require moisture for setting (e.g. AH Plus), whereas a gypsum (GS) mold with a cavity with a diameter of 10 mm and a height of 1 mm should be used for sealers that require moisture for setting, such as CSBC sealers.^18^

The molds correspond to the root canal interior; therefore, the setting time of all sealers must be measured using the same standardized molds. However, to our knowledge, there is no standardized mold that has been reported and the comparison of setting time of sealers using both SS and GS mold has not been investigated yet.

Thus, the aim of this experimental study was to compare the setting times of four CSBC sealers and epoxy resin-based sealers in SS and GS molds. The hypothesis of this study was that the setting times measured in molds made of different materials would be the same.

## Materials and methods

In this experimental study, we investigated conventional epoxy resin-based sealers and four commercially available brands of CSBC sealers: CeraSeal (CS; Meta Biomed, Cheongju, Korea), EndoSeal TCS (ES; Maruchi, Wonju, Korea), One-Fil (OF; Mediclus, Cheongju, Korea), and Well-Root ST (WR; Vericom, Chuncheon, Korea). These CSBC sealers were compared to the gold standard root canal sealer, AH Plus (AP; Dentsply DeTrey, Konstanz, Germany), an epoxy resin-based sealer. The chemical compositions of the root canal sealers investigated in this study are presented in Table 1.

**Table 1:** Chemical compositions of the root canal sealers investigated in the present study.

Sealer		Components	
Epoxy Resin-based sealer	AH Plus (AP)	Paste A	Paste B
		Bisphenol-A epoxy resin	Dibenzyldiamine
		Bisphenol-F epoxy resin	Aminoadamantane
		Calcium tungstate	Tricyclodecanediamine
		Zirconium oxide	Calcium tungstate
		Silica	Zirconium oxide
		Iron oxide pigments	Silica
			Silicone oil
Calcium Silicate-based sealer	EndoSeal TCS (ES)	Tricalcium silicate	
		Zirconium dioxide	
		Dimethyl sulfoxide	
		Thickening agent	
	One-Fil (OF)	Calcium aluminosilicate compound	
		Zirconium oxide	
		Hydrophilic polymer (thickening agent)	
	CeraSeal (CS)	Calcium silicates	
		Zirconium oxide	
		Thickening agent	
	Well-Root ST (WR)	Calcium aluminosilicate compound	
		Zirconium oxide	
		Filler	
		Thickening agent	

AP sealer was selected as a control because of its reputation as a gold standard for studies on sealers, while the remaining four types of CSBC sealers were compared in two types of molds made of SS and GS.^19^, ^20^, ^21^ All samples were analyzed before the expiration dates established by the manufacturers. Setting time was evaluated based on the ISO 6876/2012.^14^^,^^18^ Based on these guidelines, an SS mold with an internal diameter of 10 mm and height of 2 mm is recommended to measure the setting time of the AP sealer, as it does not require moisture for setting. However, CSBC sealers require moisture for setting, and since gypsum contain water and can be moist, GS molds (complying with Type 2 of ISO 6873) incorporating a cavity with a diameter of 10 mm and height of 1 mm are recommended.^22^

In this study, for the five types of root canal sealers, both SS and GS molds were used to verify the differences between the setting time of each sealer specified by the manufacturer and the setting time, depending on the type of mold.

**Figure 1:**
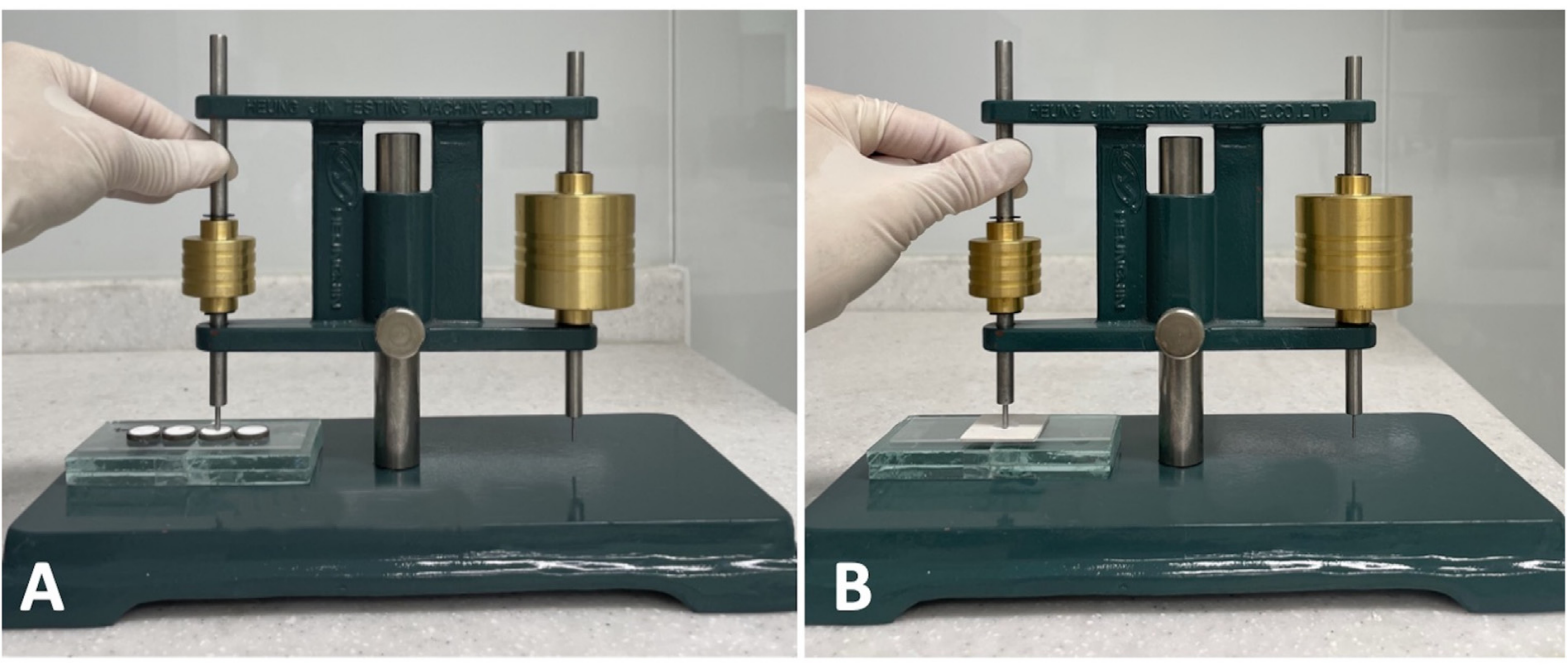
Set-up for setting time measurement using (A) stainless-steel mold and (B) gypsum mold with Gilmore needle.

Each sealer was placed in SS and GS molds on a glass slide. Sealer samples (n = 10 per group) were stored in an incubator (Changshin-lab C-IN Incubator; Pocheon, Korea) with 95% humidity at 37 °C.^14^^,^^18^^,^^21^ A Gilmore needle with a total weight of 100 g and a flat end of diameter 2.0 mm was carefully placed vertically against the sealer (Fig. 1). If an indentation was visible, the needle was raised to clean, and this process was repeated until the setting time of each sealer was measured. The setting time was measured every 5-min using a Gilmore needle starting 1 h before the setting time specified by the manufacturer. The final setting time was recorded when the needle no longer indented the sealer surface.

Statistical analysis was performed using two-way analysis of variance and Tukey parametric tests after evaluating the normality of data. The significance level was set at 5%.

## Results

The setting times of each sealer under the different test conditions are summarized in Table 2. Two-way ANOVA revealed that the setting time of all sealers in the GS mold was significantly shorter than that in the SS mold (*P* < 0.05). AP showed the longest setting time and OF showed the shortest setting time, which was significantly different from other sealers, regardless of the type of mold (*P* < 0.05). Post hoc comparison revealed no significant difference in the setting times of, ES, and CS using GS molds (*P* > 0.05). However, AP and WR showed significant differences compared to other CSBC sealers in setting time when using GS molds. AP showed the longest setting time and WR showed the second longest setting time using GS molds among five types of sealers. In addition, significant differences were found between the types of sealers and molds (*P* < 0.05).

**Table 2:** Setting time (min) of epoxy resin-based sealer (AP) and calcium silicate-based sealers under different test conditions (mean ± standard deviation).

	Types of molds	
Types of sealers	Stainless-steel mold∗	Gypsum mold

AH Plus	1108.08 ± 21.75^A^	535.42 ± 11.77^a^
EndoSeal TCS	919.29 ± 12.07^B^	91.67 ± 13.03^c^
One-Fil	286.67 ± 14.67^E^	90.71 ± 16.74^c^
CeraSeal	326.25 ± 24.23^D^	106.25 ± 13.67^bc^
Well-Root ST	415.94 ± 15.31^C^	264.58 ± 14.53^b^

∗: Setting time using stainless-steel mold was significantly longer than using gypsum mold (*P* < 0.05).

^A,B,C,D,E^: Different uppercase letters indicate significant differences depending on the types of sealers using stainless-steel mold (*P* < 0.05).

^a,b,c^: Different lowercase letters indicate significant differences depending on the types of sealers using gypsum mold (*P* < 0.05).

## Discussion

With the development of bioceramic technology, CSBC sealers have become widely used in endodontics. CSBC sealers consist of a bioceramic material that can be applied directly to the root canal using a paste-based technique. The sealers come as premixed, ready-to-use materials, stored in an airtight syringe. This permits their direct application into the canals, where they absorb moisture during the setting reaction, allowing slow setting without the need for a mixing procedure.^23^ The paste-based technique is considered less operator-dependent and potentially less damaging to the root canal dentin.^24^ In addition, CSBC sealers show good flowability, making it possible to fill even narrow root canals that are difficult to access.

Calcium silicate, the main ingredient of these sealers, forms calcium hydroxide crystals while absorbing moisture from the tissues surrounding the root canal. Calcium hydroxide crystals have high pH and inhibit the growth of bacteria inside root canals, and ensure the success of endodontic treatment by sealing the gap between the gutta-percha filling material and dentin in the root canal. In endodontics, epoxy resin-based sealers, which do not require moisture for setting, have been regarded as conventional sealers for a long time.^13^^,^^18^ However, CSBC sealers require moisture for setting; and this difference was the basis of our study to compare the setting times of both the epoxy resin-based sealer and CSBC sealers under two different test conditions.

According to recent ISO standards, an SS ring mold with an internal diameter of 10 mm and height of 2 mm should be used for sealers that do not require moisture for setting (ex. AH Plus), whereas a GS mold (complying with Type 2 of ISO 6873) incorporating a cavity with a diameter of 10 mm and height of 1 mm should be used for sealers that require moisture for setting.^22^ The SS mold simulates an environment that does not contain any moisture, because SS is hydrophobic material which does not react with moisture. GS mold, meanwhile, simulates an environment that contains moisture since the gypsum powder is known to have somewhat irregular shape and porous structure.^25^ In other words, gypsum itself has the property of absorbing and containing water or moisture and it appears to be much more hydrophilic than stainless-steel. Therefore, the SS mold corresponds to a completely dry canal and the GS mold corresponds to a moist canal. In this experimental study, two-way ANOVA analysis revealed that the setting time of all sealers in the GS mold was significantly shorter than that in the SS mold (*P* < 0.05).

Some studies have used type IV plaster molds (Durone IV Salmon; Dentsply, Petropolis, RJ, Brazil) with a diameter of 10 mm and a height of 1 mm when measuring the epoxy resin-based sealer AH Plus. These studies found setting times which were approximately 150-min shorter than the setting time of AH Plus using GS molds in the present study.^21^ Moreover, some studies used SS molds of the same size as in the present study, and showed significantly shorter setting times than those calculated in the present study.^13^ However, since these studies measured the setting time of sealers using an SS mold, the results should be interpreted carefully.

It is virtually impossible to set a completely dry condition of the root canal, regardless of how meticulously the inside of the canal is dried using paper points before performing canal filling. In most cases, dentinal tubules of the root canal contain moisture. Therefore, it is predicted that part of the current ISO standards, in which the setting time should be measured using an SS mold for a sealer that does not require moisture for setting, should be corrected by measuring the setting time using GS molds.

Moreover, some studies have shown prolonged setting time of CSBC sealers in vitro, which could be explained by the absence of tissue fluids during setting. *In vivo*, the presence of tissue fluid inside the tubules of canals may be the main source of the moist environment during the hydration reaction.^26^ In addition, some studies used a Vicat needle of 300 g total weight rather than a Gilmore needle of 100 g, which indicates that we should consider not only the types of molds, but also the types of needles, when comparing the setting time values of several studies.^14^^,^^15^

Setting time is primarily measured as a control to test the stable behavior of a product; however, this variable can fluctuate depending on the constituent components, particle sizes, ambient temperature, and relative humidity.^27^ In addition, the reason for the difference in the numerical values from other studies in the setting time measurement may be influenced by the subjectivity of the experimenter. When measuring the setting time of each sealer, we carefully lowered the Gilmore-type indenter vertically on the horizontal surface of the sealer. If an indentation was visible, the needle was raised, the needle tip was cleaned, and the needle was lowered to a new position on the surface of the sealer until the indentations ceased to be visible. Since the subjectivity of the experimenter is a regulating factor of the judgement that the indentation is sufficiently lost, differences in the result value with respect to the setting time of the sealers are inevitable. In addition, the setting time may be quite different depending on the time at which we placed the Gilmore needle on the sealer surface. If the Gilmore needle is placed too frequently, the setting of the sealer may be delayed by damage of the sealer surface by the indenter. Therefore, we should determine the time at which we place the Gilmore needle in order to avoid disturbing the settings.

In this experiment, molds with a variety of different types of sealers were stored in an incubator at a humidity of 95% and a temperature 37 °C. This can be considered a set condition because the inside of the root canal is a warm, moist environment. However, the exact value of the degree of moisture inside the root canal has not yet been calculated, and additional research is needed to set the humidity to 95% or higher in an in vitro study.

Under the limitation of this study, the results indicate that CSBC sealers require moisture for setting, and a lack of moisture can significantly delay setting time. The setting time of CSBC sealers should be measured using gypsum molds, as reported in the ISO standards. Measuring setting time using a GS mold for CSBC sealers, as well as epoxy resin sealers, should be performed to maintain clinical relevance.

## Declaration of competing interest

The authors have no conflict of interest relevant to this article.
